# Assessment of Mediastinal Tumors Using SUV_max_ and Volumetric Parameters on FDG-PET/CT

**DOI:** 10.22038/aojnmb.2016.7996

**Published:** 2017

**Authors:** Takahiro Morita, Mitsuaki Tatsumi, Mana Ishibashi, Kayako Isohashi, Hiroki Kato, Osamu Honda, Eku Shimosegawa, Noriyuki Tomiyama, Jun Hatazawa

**Affiliations:** 1 Department of Nuclear Medicine and Tracer Kinetics, Graduate School of Medicine, Osaka University, Suita, Japan; 2 Department of Radiology, Osaka University Hospital, Suita, Japan; 3 Department of Radiology, Graduate School of Medicine, Osaka University, Suita, Japan; 4 Department of Molecular Imaging in Medicine, Graduate School of Medicine, Osaka University, Suita, Japan

**Keywords:** FDG-PET/CT, Mediastinal tumor, Metabolic tumor volume, Total lesion glycolysis

## Abstract

**Objective(s):**

This study aimed to evaluate the role of pretreatment SUV_max_ and volumetric FDG positron emission tomography (PET) parameters in the differentiation between benign and malignant mediastinal tumors. In addition, we investigated whether pretreatment SUV_max_ and volumetric FDG-PET parameters could distinguish thymomas from thymic carcinomas, and low-risk from high-risk thymomas.

**Methods:**

This study was conducted on 52 patients with mediastinal tumors undergoing FDG-PET/CT. Histological examination indicated that 29 mediastinal tumors were benign, and 23 cases were malignant. To obtain quantitative PET/CT parameters, we determined the maximum standardized uptake value (SUV_max_), volumetric parameters, metabolic tumor volume (MTV), and total lesion glycolysis (TLG) for primary tumors using SUV_max_ cut-off value of 2.5. SUV_max_, MTV and TLG of benign and malignant tumors were compared using the Mann-Whitney U test. Moreover, receiver-operating curve (ROC) analysis was applied to identify the cut-off values of SUV_max_, MTV and TLG for the accurate differentiation of benign and malignant tumors. SUV_max_, MTV and TLG were compared between thymomas and thymic carcinomas, as well as low-risk and high-risk thymomas.

**Results:**

Mean SUV_max_, MTV and TLG of malignant mediastinal tumors were significantly higher compared to benign tumors (P<0.001). Sensitivity, specificity, accuracy, positive predictive value, and negative predictive value of SUV_max_ were 78.2%, 86.2%, 82.6%, 81.8%, and 83.3%, respectively. These values were estimated at 82.6%, 96.6%, 90.4%, 95%, and 87.5% for MTV and TLG, respectively. Additionally, optimal cut-off values for the differentiation of benign and malignant mediastinal tumors were determined at 4.2 and 22.3 mL and 79.7 g for SUV_max_, MTV and TLG, respectively. Mean SUV_max_, MTV and TLG of thymic carcinomas were significantly higher compared to thymomas (P<0.01), while no significant differences were observed in the mean quantitative parameters between low-risk and high-risk thymomas.

**Conclusion:**

Although SUV_max_, MTV and TLG could not distinguish between low-risk and high-risk thymomas, these parameters might be able to differentiate benign tumors from malignant mediastinal tumors noninvasively. These parameters could be used to distinguish between thymomas and thymic carcinomas as well. Therefore, FDG-PET/CT parameters seem to be accurate indices for the detection of malignant mediastinal tumors.

## Introduction:

Mediastinal tumors span a wide histopa-thological and radiological spectrum. Although more than two-third of these tumors are benign, malignancies are likely to occur in the anterior compartment (1).

Thymomas are the most prevalent anterior mediastinal tumors encountered in the anterior mediastinum. According to the histological criteria published by the World Health Organization (WHO) in 2004, thymomas are of three histopathological types, including low-risk thymomas (types A, AB and B1), high-risk thymomas (types B2 and B3), and thymic carcinomas (type C) (2-5).

Differentiation of benign mediastinal tumors from malignancies is essential to determining therapeutic options and assessing the prognosis. Several studies have denoted that mediastinal tumors have characteristic CT and MRI findings (1, 6-8). Nonetheless, modalities such as CT or MRI could not distinguish between benign and malignant tumors accurately (9-13).

FDG positron emission tomography (PET) imaging is routinely performed for the staging, restaging, treatment planning and follow-up of various solid tumors (14). In the analysis of the prognostic capability of FDG-PET/CT, one of the most common parameters is the maximum standardized uptake value (SUV_max_) of the primary tumor.

Recently, two 3D-FDG parameters of metabolic tumor volume (MTV) and total lesion glycolysis (TLG) have been proposed as the imaging biomarkers of potential interest for diagnostic and prognostic assessment of cancer patients (15-18). MTV signifies the volume of the tumor tissue, demonstrating increased FDG uptake above a certain threshold. TLG is obtained by multiplying the MTV by the mean SUV.

With the rapid expansion of clinical PET/CT, more opportunities are available to evaluate mediastinal tumors through this approach. However, limited studies have investigated the value of FDG-PET/CT for the evaluation of mediastinal tumors using SUV_max_ (19, 20). Therefore, this study aimed to evaluate the role of pretreatment SUV_max_ and volumetric FDG-PET parameters in the differentiation of benign and malignant mediastinal tumors. In addition, we assessed whether pretreatment SUV_max_ and volumetric FDG-PET parameters could distinguish between thymomas and thymic carcinomas, as well as low-risk and high-risk thymomas.

## Methods

### Patients

This retrospective study was conducted on 52 patients with mediastinal tumors, including 25 men and 27 women within the age range of 20-83 years (mean age: 51.4±16.6 years). Study protocol was approved by the Ethics Committee of Osaka University Hospital, Japan (January 19, 2015).

Inclusion criteria were patients with a mediastinal tumor based on CT or MRI, who had received no biopsy, treatment or surgery. Selected patients had received FDG-PET/CT chest examinations during April 2007-December 2015.

In total, 52 mediastinal tumors were pathologically confirmed via surgical excision or percutaneous biopsy. Samples consisted of thymic epithelial tumors (n=20), neurogenic tumors (n=5), thymic cysts (n=4), mature teratomas (n=3), thymic hyperplasia (n=2), solitary fibrous tumor (n=1), parathyroid adenoma (n=1), malignant lymphomas (n=9), thymic carcinomas (n=5), well-differentiated liposarcoma (n=1), and multiple myeloma (n=1) (Table 1).

**Table 1:** Mean, standard deviation and range of SUV_max_, MTV and TLG for mediastinal tumors

Histological type	Lesions (N)	SUV_max_	MTV (mL)	TLG (g)

		Mean±SD (range)	Mean±SD (range)	Mean±SD (range)
Low-risk thymoma	13	3.0±1.2 (1.8-6.5)	12.3±32.9 (0-125)	45.0±128.4 (0-487.5)
Neurogenic tumor	5	2.9±1.1 (2.2-5)	2.3±4.4 (0-11.4)	7.2±14.2 (0-35.5)
Thymic cyst	4	1.0±0.3 (0.7-1.5)	0	0
Mature teratoma	3	3.3±1.4 (1.8-5.2)	3.0±4.0 (0-8.6)	9.0±12 (0-25.9)
Thymic hyperplasia	2	4.7±0.8 (3.9-5.4)	12.8±1.9 (10.9-14.6)	39.2±8.2 (31-47.3)
Solitary fibrous tumor	1	1.3	0	0
Parathyroid adenoma	1	2	0	0
Malignant lymphoma	9	11.1±4.3 (4.7-18.5)	130.1±73.7 (34-291)	692.7±524.4 (125.2-1920.6)
High-risk thymoma	7	3.3±1.5 (1.6-5.6)	16.6±22.2 (0-62.7)	55.5±74.9 (0-213.9)
Thymic carcinoma	5	8.7±3.7 (5.4-15.1)	109.6±44.3 (27.7-249)	467.3±294.4 (88.7-918.8)
Well-differentiated liposarcoma	1	3.3	29.5	79.7
Multiple myeloma	1	7.8	64.5	238

High-risk thymomas and thymic carcinomas were considered clinically malignant, whereas low-risk thymomas were considered clinically benign. In total, 29 benign and 23 malignant tumors were examined in this study.

### PET/CT examination

Initially, selected patients were asked to fast for a minimum of four hours prior to FDG administration. Each patient was intravenously injected with 3.7 MBq/kg of FDG in the antecubital vein followed by PET/CT, which started 60 minutes after the injection. In this process, we used an integrated PET/CT unit scanner (Gemini GXL, Philips).

Scanning was performed from the top of the skull to the mid-thighs of the patients. Acquired PET data included the three-dimensional emission scans of 11 bed positions covering the mentioned area, accompanied with a two-minute data acquisition for one bed position and ordered subset expectation maximization reconstruction with the slice thickness of 4.0 mm.

CT images were obtained before the PET scan, and CT data were used for attenuation correction and image fusion. Moreover, a CT-scan was carried out using a breath-holding technique with normal expiration from the apex level of lungs to the lower poles of kidneys. It is also noteworthy that no intravenous or oral contrast medium was used in this regard.

Irradiation voltage and current were 120 kVp and 50 effective mAs, respectively. CT detector was composed of a 16-ring alignment, and detector collimation was set at 1.5 mm. Slice thickness of the images was estimated at 5.0 mm, with a center-to-center interval of 4.0 mm.

### PET/CT evaluation

To determine the PET/CT parameters, SUV_max_, MTV and TLG were obtained for the primary tumor based on the previously described method (21). In brief, SUV_max_ represents the highest activity of a single pixel within the tumor, and MTV measures the volume of the metabolically active tumor. TLG, which is the multiplication of MTV and SUV_mean_, signifies the overall tumor burden (15).

To define the contouring margins around the tumor, SUV cut-off value was considered at 2.5 (22, 23). If SUV_max_ of a tumor was equal to or less than the determined threshold, MTV and TLG would be considered zero.

### Statistical analysis

Mean SUV_max_, MTV and TLG of benign and malignant tumors were compared using the Mann-Whitney U test. In addition, receiver-operating characteristic (ROC) analysis was used to identify the cut-off values for SUV_max_ and two 3D-FDG parameters, which accurately differentiated benign tumors from malignant tumors. Sensitivity, specificity, accuracy, and positive and negative predictive values were calculated for each threshold value.

Sensitivity, specificity and accuracy for the differentiation between benign and malignant tumors was obtained. In this respect, sensitivity was defined as the percentage of malignancies with index levels equal to or higher than both threshold levels, and specificity was defined as the percentage of benign tumors with index levels lower than both threshold levels.

McNemar’s test was used for comparison and P<0.05 was considered as significant in all comparisons. Moreover, differences in the area under the ROC curve (AUC) between SUV_max_ and two 3D-FDG parameters were assessed using the method proposed by Hanley and McNeil.

## Results

**Figure 1:**
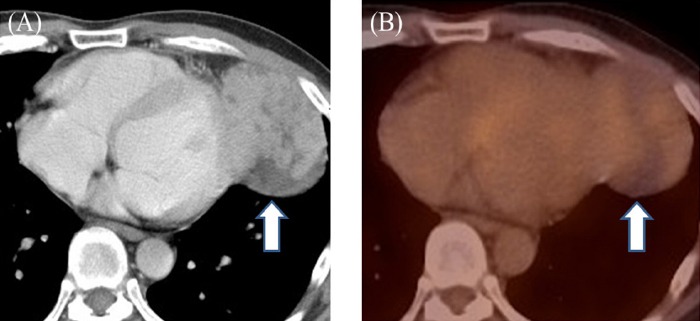
(A) Contrast-enhanced CT image shows a partially ill-defined, heterogeneous left-anterior mediastinal tumor (arrow); (B) PET/CT image shows low FDG uptake (arrow); SUV_max_, MTV and TLG estimated at 2.0, zero and zero, respectively A 44-year-old man with type B1 thymoma

**Figure 2:**
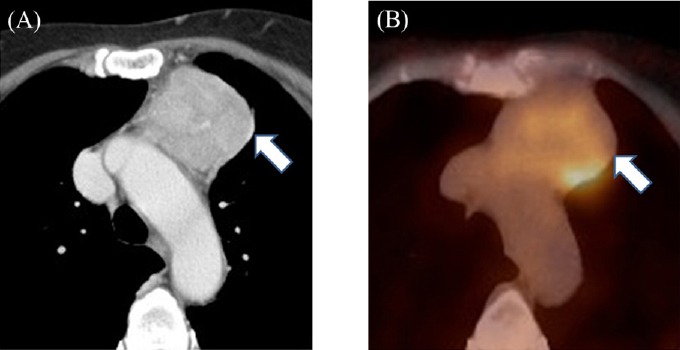
A 64-year-old woman with thymic carcinoma (A) Contrast-enhanced CT image shows a well-defined, homogeneous anterior mediastinal tumor (arrow); (B) PET/CT image shows high FDG uptake (arrow); SUV_max_, MTV and TLG estimated at 5.4, 27.7 mL and 88.7 g, respectively

Representative mediastinal tumors are depicted in figures 1 and 2.

Mean SUV_max_, MTV and TLG of the studied mediastinal tumors are presented in Table 1. Mean SUV_max_, MTV and TLG of benign mediastinal tumors were 2.7±1.4, 7.1±22.8 mL and 25.0±88.6 g, respectively.

**Figure 3:**
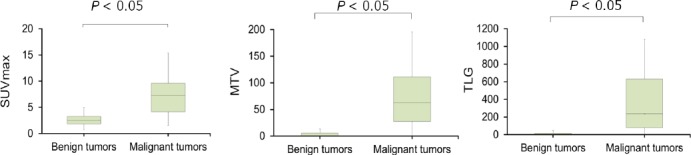
Distribution of SUV_max_, MTV and TLG between benign and malignant mediastinal tumors

In malignant mediastinal tumors, these values were determined at 7.7±4.7 ml, 83.9±78.6 mL and 403.3±460.1 g, respectively. According to our findings, mean SUV_max_, MTV and TLG of malignant mediastinal tumors were significantly higher compared to benign tumors (Figure 3).

**Figure 4:**
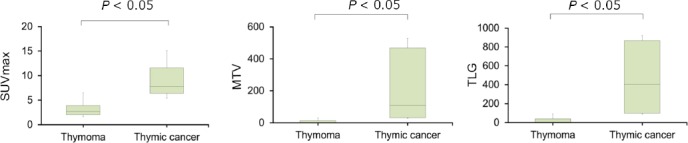
Distribution of SUV_max_, MTV and TLG between thymomas and thymic carcinomas

In this study, mean SUV_max_, MTV and TLG of thymomas were 3.1±1.3, 13.8±29.6 mL and 48.7±112.7 g, respectively. In thymic carcinomas, these values were estimated to be 8.7±1.7 ml, 109.6±40.1 mL and 467.3±173.4 g, respectively. Results of this study indicated that mean SUV_max_, MTV and TLG of thymic carcinomas were significantly higher compared to thymomas (Figure 4).

**Figure 5:**
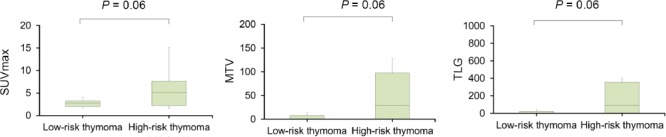
Distribution of SUV_max_, MTV and TLG between low-risk and high-risk thymomas

Mean SUV_max_, MTV and TLG of low-risk thymomas were determined to be 3.0±1.2 ml, 12.3±32.9 mL and 45.0±128.4 g, respectively. In high-risk thymomas, these values were 3.3±1.5, 16.6±22.2 mL and 55.5±74.9 g, respectively. No significant differences were observed in the mean quantitative parameters between low-risk and high-risk thymomas (Figure 5).

**Figure 6:**
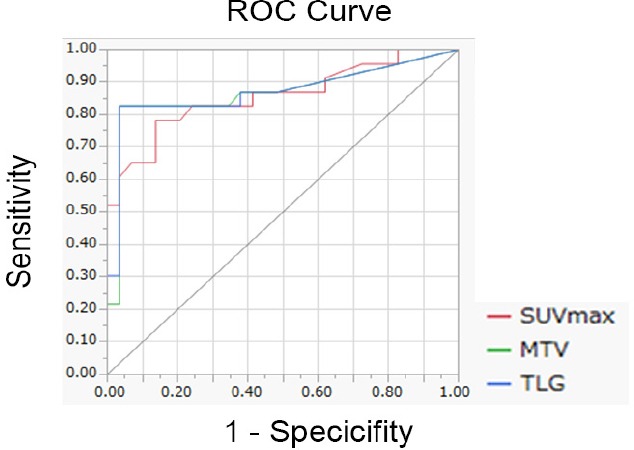
ROC curve for differentiation between benign and malignant mediastinal tumors

Results of ROC analysis for the differentiation of benign and malignant mediastinal tumors are depicted in Figure 6. Accordingly, AUCs of SUV_max_, MTV and TLG were 0.857, 0.867 and 0.869, respectively, and no significant differences were observed in these values between benign and malignant tumors.

Diagnostic capability for the differentiation between benign and malignant tumors is summarized in Table 2. According to the information in this table, optimal cut-off values for distinguishing between benign and malignant mediastinal tumors were 4.2 ml, 22.3 mL and 79.7 g for SUV_max_, MTV and TLG, respectively. Furthermore, no significant differences were observed in the sensitivity, specificity and accuracy of SUV_max_, MTV and TLG.

**Table 2:** Ability of SUV_max_, MTV and TLG for histological differentiation of benign and malignant mediastinal tumors

	AUC	Cut-off	Sensitivity (%)	Specificity (%)	Accuracy (%)	PPV (%)	NPV (%)
SUV_max_	0.857	4.2	78.2	86.2	82.6	81.8	83.3
MTV	0.867	22.3 mL	82.6	96.6	90.4	95	87.5
TLG	0.869	79.7 g	82.6	96.6	90.4	95	87.5

**PPV:** positive predictive value; **NPV:** negative predictive value; *Significant difference with SUV_max_ according to McNemar’s test (P<0.05)

## Discussion

According to the results of the present study, SUV_max_, MTV and TLG of FDG-PET/CT had high sensitivity and specificity for the detection of malignant mediastinal tumors. Although SUV_max_, MTV and TLG could not distinguish between low-risk and high-risk thymomas, they were able to differentiate thymomas from thymic carcinomas.

Our findings indicated that FDG-PET/CT had high sensitivity and specificity for the detection of mediastinal malignancies. As some surgeons advocate tumor resection without biopsy or treatment, pretreatment FDG-PET/CT seems to be a proper modality to further characterize mediastinal tumors and reduce unnecessary invasive procedures in cases with low FDG uptake.

In a study in this regard, Kubota et al. reported that mean SUV_max_ of malignant mediastinal tumors was significantly higher compared to that of benign tumors. Furthermore, the cut-off value to accurately distinguish between benign and malignant mediastinal tumors was a differential uptake ratio (synonymous with SUV), which was estimated at approximately 3.5 (19).

According to the results of the current research, mean SUV_max_ for malignant tumors was significantly higher compared to that of benign tumors with the optimal SUV_max_ cut-off value of 4.2.

Thymomas are slow-growing masses, which mainly originate from thymic epithelial cells, while thymic carcinomas behave aggressively and are associated with a poor prognosis. FDG-PET/CT is reportedly helpful in the diagnosis of these masses, as several studies have emphasized that SUV_max_ could distinguish thymomas from thymic carcinomas (24-26). This is consistent with the results of the present study. In addition to SUV_max_, MTV and TLG were found to be able to differentiate thymomas from thymic carcinomas in our study.

However, ability of ^18^F-FDG-PET/CT to distinguish between high-risk and low-risk thymomas remains a matter of debate. In this regard, some studies have denoted that SUV_max_ could differentiate between low-risk and high-risk thymomas (24, 27, 28), whereas other studies have proposed conflicting results (29, 30). Due to this discrepancy, we aimed to determine whether SUV_max_, MTV and TLG could distinguish between low-risk and high-risk thymomas.

SUV_max_ represents the highest point of metabolic activity, thereby reflecting the most biologically aggressive area rather than the whole tumor. On the other hand, MTV signifies the volume of the tumor tissue with an increased FDG uptake over a certain threshold, while TLG delineates the overall tumor burden of FDG uptake. Therefore, we hypothesized that MTV and TLG might be effective in the differentiation of low-risk and high-risk thymomas. However, our findings indicated that MTV, TLG and SUV_max_ could not distinguish between these lesions, which could be due to the small patient population and heterogeneous distribution of parameters in patients with high-risk thymomas in the present study.

One of the limitations of the current research was the wide histopathological spectrum of mediastinal tumors. As such, a clinical differential diagnosis should include other mediastinal tumors, while we only evaluated 12 types of mediastinal tumors. With this background in mind, it is recommended that further investigation be conducted on larger sample sizes, so that each case would represent a specific pathological entity.

Moreover, since SUV is affected by various biological and technical factors (e.g., body weight, serum glucose level, reconstruction methods and noise), it is suggested that SUV cut-off value be interpreted discreetly (31, 32).

## Conclusion

Although SUV_max_, MTV and TLG could not differentiate between low-risk and high-risk thymomas, these parameters might be able to distinguish between benign and malignant mediastinal tumors noninvasively, as well as thymomas and thymic carcinomas. In conclusion, these FDG-PET/CT parameters appear to be proper tools for the accurate detection of malignant mediastinal tumors.
